# Variations of Gastrocolic Trunk of Henle and Its Significance in Gastrocolic Surgery

**DOI:** 10.1155/2018/3573680

**Published:** 2018-06-06

**Authors:** Yuan Gao, Yun Lu

**Affiliations:** ^1^Department of General Surgery, The Affiliated Hospital of Qingdao University, Qingdao, China; ^2^Shandong Key Laboratory of Digital Medicine and Computer Assisted Surgery, Qingdao, China

## Abstract

Due to the increasing incidence of gastrointestinal (GI) tumors, more and more importance is attached to radical resection and patients' survival, which requires adequate extent of resection and radical lymph node dissection. Blood vessels around the gastrointestinal tract, as anatomical landmarks for tumor resection and lymph node dissection, play a key role in the successful surgery and curative treatment of gastrointestinal tumors. In the isolation of subpyloric area or hepatic flexure of the colon for gastrectomy or right hemicolectomy, lymph node dissection and ligation are often performed at the head of the pancreas and superior mesenteric vein, during which even a minor inadvertent error may lead to unwanted bleeding. Among these blood vessels, the venous system composed of Henle's trunk and its tributaries is the most complex, which has a direct influence on the outcome and postoperative recovery of the patients. There are many variations of Henle's trunk, with complicated courses and various locations, attracting more and more researchers to study it and tried to analyze the influence of its variations on gastrointestinal surgeries. We characterized various variants and tributaries of Henle's trunk using autopsy, vascular casting, 3D CT reconstruction, intraoperative anatomy, and Hisense CAS system and summarized and analyzed the tributaries of Henle's trunk, to determine its influence on GI surgeries.

## 1. Introduction

Resection of tumor along blood vessels and lymph node dissection has been the basic procedures in the surgical treatment of GI tumors. Therefore, understanding of the anatomy and variations of the blood vessels determines the result of the surgery and prognosis of the patients. In recent years, Henle's trunk has attracted more and more attention because of its special anatomical position and the role as an anatomical landmark in GI surgeries, and more and more studies were done on it. Various methods have been employed to identify the course and variations of Henle's trunk usage, suggesting its clinical importance and variability and complexity. This study reviewed the definition, construction, variations, and significance in gastrocolic surgeries of Henle's trunk.

## 2. Definition and Construction of Henle's Trunk

The concept of gastrocolic venous trunk was first proposed by Henle [1] in 1868. It is a venous trunk, later known as Henle's trunk or Henle's gastrocolic trunk (GTH), connecting part of the blood supply to the stomach and colon, which is formed by the convergence of the stomach-draining right gastroepiploic vein (RGEV) and the colon-draining superior right colic vein (SRCV), and drains into the superior mesenteric vein (SMV) at the inferior border of the pancreas. After nearly half a century, Descomps et al. [2] completed the definition of Henle's trunk by introducing the anterior superior pancreaticoduodenal vein (ASPDV), which made Henle's trunk venous trunk formed by 3 veins. With the increasing intention to Henle's trunk, more and more studies on it have been done using new approaches, from autopsy and vascular casting to the intraoperative anatomy and preoperative 3D CT reconstruction, providing new insight into the construction and variations of Henle's trunk.

## 3. Definition of Vein Tributaries Coming from the Colon

There are several variations in the formation of the GTH that depend on the number of tributaries of the right colic, which are mainly classified as bipod, tripod, and tetrapod. The right colic vein (RCV) and middle colic vein (MCV) are defined as the tributaries from the marginal veins of the ascending and transverse colon, respectively. When more than two RCVs or MCVs are present, the thickest vein is defined as the main vein, while the thinner vein is called the accessory vein. The SRCV is defined as the tributary from the marginal veins of the hepatic flexure.

## 4. Studies on Variations of Henle's Trunk

### 4.1. Autopsy and Vascular Casting

Studies on blood vessel variations using autopsy and vascular casting have obtained accurate results, but failed to identify some rare variants due to small samples. Results of nearly a hundred studies on Henle's trunk using autopsy and vascular casting are summarized in Table 1, which showed that the occurrence of Henle's trunk varied from 69% to 100%, suggesting the absence of Henle's trunk in many people, due mainly to that RGEV and colic veins did not converge. Among most common types of Henle's trunk are those formed by RGEV, ASPDV, and a colic vein. In the studies by Yamaguchi et al. [3] and Ignjatovic et al. [4], the accessory right colic vein (aMCV) served as the colic tributary in a very large proportion of cases, but Jin et al. [5] and Ignjatovic et al. [6] showed that SRCV or RCV, especially the former, were the most common tributaries. In studies before the 21st century, including when Henle proposed the gastrocolic venous trunk, SRCV or RCV were most frequently reported. But in recent years, more types of Henle's trunk were identified with the induction of aMCV. What is more, Ignjatovic et al. [6] identified the anterior inferior pancreaticoduodenal vein (AIPDV), though rarely seen, as a tributary of Henle's trunk.

### 4.2. Intraoperative Anatomy

Surgical procedures are actually based on the anatomy of organs and vasculature, and the anatomy of the complex vasculature, in particular, has a direct influence on the success of surgery. Henle's trunk, a both relatively undiversified but also variable venous trunk, is a key to many surgeries, especially those in the stomach, pancreas, and right side of the colon, which involves the three most common tributaries of Henle's trunk: RGEV, ASPDV, and colic veins. Intraoperative studies on Henle's trunk were summarized in Table 2, which showed the presence of Henle's trunk in 45.9% (Lange et al. [7]) to 88.6% of all subjects. In most studies, SRCV was identified as a major tributary of Henle's trunk, though Alsabilah et al. [8] reported Henle's trunk formed by RGEV and ASPDV only, without colic veins, in 58.1% of all patients, which was rarely seen in existing studies, and whether it was a type of Henle's trunk remains to be discussed.

### 4.3. 3D CT Reconstruction

In the 21st century, rapid development of radiology, especially 3D CT reconstruction, enables the establishment of 3D image of the lesion preoperatively. The 3D reconstruction of blood vessels enables the visualization of the patients' vasculature, allowing surgeons to predict difficulties in the procedure and develop appropriate treatment plans. Recent studies on Henle's trunk variations using 3D CT reconstruction are summarized in Table 3. In these studies, the sample size was usually large because of the noninvasive and easy-to-use technology, and Henle's trunk was identified in 77.5% to 87.7% of the subjects, and more types of variations were observed. However, there were some false-positive results that should be excluded, since it was a simulative imaging of the vessels. Sakaguchi et al. [10] did not mention ASPDV as a tributary of Henle's trunk, which does not mean the absence of ASPDV. According to previous studies, it is not possible that ASPDV is not part of ASPDV Henle's trunk in such a large number of patients. Ogino et al. [11] and Miyazawa et al. [12] reported that the construction of Henle's trunk is fixed, with RGEV and ASPDV, as well as variations of colic veins, of which SRCV and RCV were the most important tributaries of Henle's trunk.

### 4.4. Hisense Computer-Assisted Surgery (CAS) System

CAS system, a virtual stereotactic surgical system developed by our hospital in collaboration with Hisense group, can obtain virtual images of the patients' lesions preoperatively, providing basis for the treatment and accurate navigation for the surgery. Recently, Hisense CAS system is used to obtain images of Henle's trunk for the study of its variations, in order to guide the development of surgical plans. Our study included a total of 120 patients, with Henle's trunk identified in 102 (85.0%) of them. We classified Henle's trunk into 2 types and 10 subtypes, and the most common one is those formed by SRCV, as a colic tributary, and GEV and ASPDV, which is found in 31.4% of all patients (Table 4).

### 4.5. Summary of Variations of Henle's Trunk

**Figure 1:**
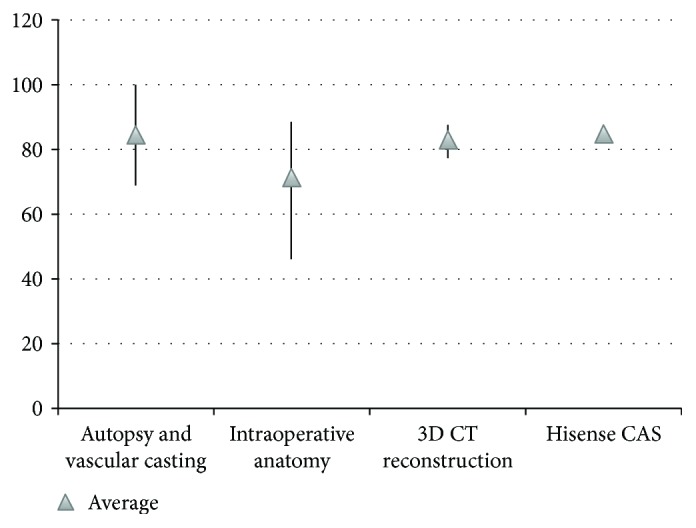
Frequency of Henle's trunk identified by various study methods.

**Figure 2:**
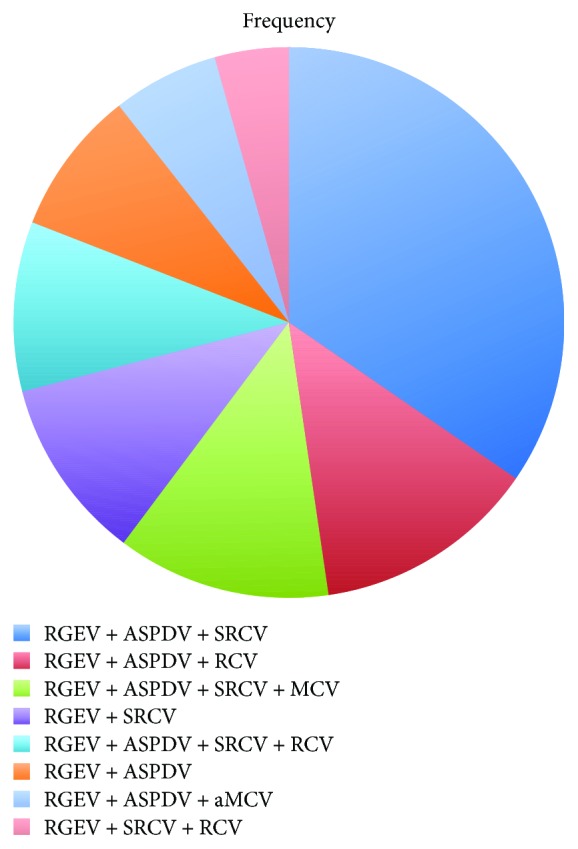
Occurrence of various variations of Henle's trunk.

In studies using all of the four approaches, expected for intraoperative anatomy, Henle's trunk was identified in more than 80% of patients, suggesting a stable existence of Henle's trunk in the human body (Figure 1). Frequency of Henle's trunk identified by autopsy, vascular casting, and intraoperative anatomy varied greatly from 45.9% to 100%. In the study of Henle's trunk using 3D CT reconstruction, the frequency of Henle's trunk is basically invariable, which is similar with those obtained by Hisense CAS system. In addition, the composition of the Henle's trunk was analyzed, and of the 936 patients with Henle's trunk included, 503 demonstrated common types (53.7%) (Figure 2), and the rest 46.3% had rare types. Among the common types, RGEV + ASPDV + SRCV was most frequently seen, in 34.6%, followed by RGEV + ASPDV + RCV, in 13.1%.

## 5. Effect of Variations of Henle's Trunk on Surgery for Gastric Cancer and Dissection of Number 6 Lymph Nodes

Lymph node metastasis is the main route of metastasis of gastric cancer, and radical dissection of lymph node during the surgery had a direct influence on the outcome and prognosis [13, 14]. The Japanese Gastric Cancer Association (JGCA) defined the subpyloric lymph nodes anterior to the pancreas as number 6 lymph nodes, which extend down the right gastroepiploic artery (RGEA) until the convergence of RGEV and ASPDV [15]. Later, number 6 lymph nodes were divided into 3 subgroups based on the course of the blood vessels, with those distributed along the RGEA, RGEV, and subpyloric vessels defined as number 6a, number 6v, and number 6i, respectively [16, 17].  Table 5 summarized the number 6 lymph node metastasis of patients with gastric cancer, which showed a total metastasis rate of 5.7% to 30.6%, and an increased rate in lower stomach cancer, with the highest being 37.0%. Therefore, dissection of number 6 lymph nodes is imperative in the radical treatment of gastric cancer, especially radical resection of distal stomach. In the dissection of number 6 lymph nodes, the right gastroepiploic vessels serve as an important landmark, especially RGEV, which has variable courses and multiple communication and convergences, causes some difficulty to the dissection.  Table 6 showed that RGEV serving as a tributary of Henle's trunk was most frequently seen (in up to 100%), followed by convergence with ASPDV before draining into SMV (in 7%–18.8%), and draining into SMV directly was the least (in 6.3%–22.5%). Great care must be taken in the dissection of number 6 lymph nodes, especially in the radical treatment of gastric cancer, in order not to injure RGEV, whose bleeding may severely affect the anatomy of its root and lymph node dissection, because of its thin, fragile wall.

## 6. Effect of Variations of Henle's Trunk on Right Hemicolectomy and CME

**Figure 3:**
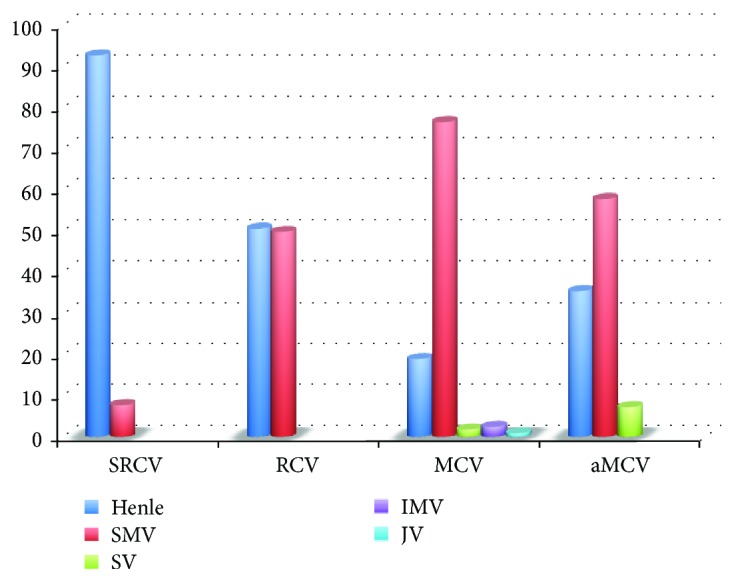
Percentage of veins receiving blood from right colon-draining veins.

Complete mesocolic excision (CME) was first proposed by Hohenberger et al. in 1992 on the basis of embryology and anatomy, which brought about a revolution in the radical treatment of colon cancer [22, 23]. CME is the extension of total mesorectal excision (TME) and involves the complete sharp isolation of visceral fascia, dissection of lymph nodes around the mesenteric artery root, and high ligation of central feeding vessel [24, 25]. Although the use of CME is still controversial until now, focusing on its anatomical layer and vascular ligation is the trend of radical treatment of colon cancer. Among the blood vessels supplying the colon, the venous system draining the right side is the most complex, with the criss-cross of SRCV, RCV, MCV, and aMCV, which, together with Helen's trunk and SMV, forms a complex 3D vascular system.  Table 7 showed significant variations of the veins draining the right side of the colon, and the absence of SRCV and RCV in some cases, possibly due to the mutual complementation of the two vessels. SRCV and RCV draining into the Henle's trunk were most frequently seen, followed by those draining into the SMV. As for MCV, there are more variations, mostly (up to 94.0%) drains into SMV, then into Henle's trunk, and few into the jejunal vein (JV), inferior mesenteric vein (IMV), and splenic vein (SV). aMCV was reported in some studies, with a high rate of absence. It mostly drains into Henle's trunk or SMV (Figure 3). Therefore, it is important that the surgeon knows well the anatomy of the variations of Henle's trunk and surrounding vessels in right hemicolectomy, to avoid unwanted bleeding and achieve better outcomes.

In conclusion, Henle's trunk, which connects the stomach and colon-draining veins, plays an important role in surgeries for the stomach and colon and shall be isolated for vascular ligation and lymph node dissection in many surgical procedures, especially after the rapid development of laparoscopic and robot-assisted surgery in recent years. However, the intraoperative anatomy of Henle's trunk is challenging because of its fixed position and variable combinations of tributaries, which make it difficult to predict its course and lead to increased incidence of bleeding and complications. Many studies have been done in recent years using various radiological approaches to analyze and summarize the variations of Henle's trunk and gained great achievements, which enables acquisition of knowledge of its tributaries and variation preoperatively, to ensure the outcome and prognosis of the patients [10–12, 27].

## Figures and Tables

**Table 1:** Variations of Henle's trunk identified by autopsy and vascular casting.

Author	Year	Case (*n*)	Frequency, *n* (%)	Type (%)
Yamaguchi et al. [3]	2002	40	40/58 (69.0)	RGEV + ASPDV + RCV (25.0)
				RGEV + ASPDV + RCV + aMCV (2.5)
				RGEV + ASPDV + MCV (17.5)
				RGEV + ASPDV + aMCV (55.0)

Ignjatovic et al. [4]	2004	10	10/10 (100.0)	RGEV + ASPDV + aMCV (90.0)
				RGEV + ASPDV + MCV (10.0)

Jin et al. [5]	2006	8	8/9 (88.9)	RGEV + ASPDV + SRCV (37.5)
				RGEV + ASPDV + SRCV + RCV (50.0)
				RGEV + ASPDV + SRCV + RCV + MCV (12.5)

Ignjatovic et al. [6]	2010	34	34/42 (81.0)	RGEV + SRCV (26.5)
				RGEV + SRCV + ASPDV or AIPDV (73.5)

RCV = right colic vein; MCV = middle colic vein; aMCV = accessory middle colic vein; SRCV = superior right colic vein; ASPDV = anterior superior pancreaticoduodenal vein; RGEV = right gastroepiploic vein; AIPDV = anterior inferior pancreaticoduodenal vein.

**Table 2:** Variations of Henle's trunk identified by intraoperative anatomy.

Author	Year	Case (*n*)	Frequency, *n* (%)	Type (%)
Lange et al. [7]	2000	17	17/37^∗^ (45.9)	RGEV + ASPDV + SRCV (82.4)
				RGEV + SRCV (17.6)

Lee et al. [9]	2016	92	92/116 (79.3)	RGEV + ASPDV + SRCV + MCV (68.5)
				RGEV + ASPDV + SRCV (31.5)

Alsabilah et al. [8]	2017	62	62/70 (88.6)	RGEV + ASPDV (58.1)
				RGEV + ASPDV + RCV (16.1)
				RGEV + ASPDV + RCV + aMCV (8.1)
				RGEV + ASPDV + RCV + MCV (3.2)
				RGEV + ASPDV + MCV (3.2)

^∗^Include 14 autopsies. RCV = right colic vein; MCV = middle colic vein; aMCV = accessory middle colic vein; SRCV = superior right colic vein; ASPDV = anterior superior pancreaticoduodenal vein; RGEV = right gastroepiploic vein.

**Table 3:** Variations of Henle's trunk identified by preoperative 3D CT reconstruction.

Author	Year	Case (*n*)	Frequency, *n* (%)	Type (%)
Sakaguchi et al. [10]	2010	79	79/102 (77.5)	RGEV + SRCV (53.2)
				RGEV + RCV (1.3)
				RGEV + MCV (2.5)
				RGEV + SRCV + RCV (19.0)
				RGEV + SRCV + MCV (12.7)
				RGEV + SRCV + RCV + MCV (11.4)

Ogino et al. [11]	2014	71	71/81 (87.7)	RGEV + ASPDV + RCV (40.8)
				RGEV + ASPDV + MCV (1.4)
				RGEV + ASPDV + RCV + MCV (31.0)
				RGEV + ASPDV + SRCV + RCV (19.7)
				RGEV + ASPDV + SRCV + RCV + MCV (4.2)
				RGEV + ASPDV + ICV + RCV + MCV (2.8)

Miyazawa et al. [12]	2015	100	100/120 (83.3)	RGEV + ASPDV (7.0)
				RGEV + ASPDV + SRCV (71.0)
				RGEV + ASPDV + SRCV + RCV or MCV (20.0)
				RGEV + ASPDV + SRCV + RCV + MCV (2.0)

RCV = right colic vein; MCV = middle colic vein; SRCV = superior right colic vein; ASPDV = anterior superior pancreaticoduodenal vein; RGEV = right gastroepiploic vein.

**Table 4:** Classification of GTH based on ASPDV and venous tributaries from the right colon.

Type of GTH	Variety of drainage vein	Frequency *n* (%)
I (gastrocolic type, GC)		33 (32.4)
Ia	RGEV + SRCV	12 (11.8)
Ib	RGEV + RCV	8 (7.8)
Ic	RGEV + SRCV + RCV	7 (6.9)
Id	RGEV + SRCV + MCV	4 (3.9)
Ie	RGEV + RCV + MCV	2 (2.0)

II (gastro-pancreatic-colic type, GPC)		69 (67.6)
IIa	RGEV + ASPDV + SRCV	32 (31.4)
IIb	RGEV + ASPDV + RCV	17 (16.7)
IIc	RGEV + ASPDV + SRCV + RCV	12 (11.8)
IId	RGEV + ASPDV + SRCV + RCV + MCV	5 (4.9)
IIe	RGEV + ASPDV + MCV	3 (2.9)

GTH = gastrocolic trunk of Henle; RCV = right colic vein; MCV = middle colic vein; SRCV = superior right colic vein; ASPDV = anterior superior pancreaticoduodenal vein; RGEV = right gastroepiploic vein.

**Table 5:** Analysis of number 6 lymph node metastasis in surgery for gastric cancer.

Author	Year	Total metastatic rate (%)	L (%)	M (%)	U (%)
Methasate et al. [18]	2010	N	37.0	41.0	10.0
Han et al. [19]	2011	12.6	18.7	7.1	1.9
Haruta et al. [16]	2013	5.7	N	N	N
Zuo et al. [20]	2014	26.4	34.0	13.9	2.0
Cao et al. [21]	2015	30.6	30.6	N	N

L = lower gastric cancer; M = middle gastric cancer; U = upper gastric cancer; N = not mentioned.

**Table 6:** Types of draining pattern of the right gastroepiploic vein.

Author	Year	Case (*n*)	Draining vein of RGEV (%)
Lange et al. [7]	2000	37	Henle's trunk (45.9)
			Flow into SMV with ASPDV (43.2)
			SMV (10.8)

Ignjatovic et al. [4]	2004	10	Henle's trunk (100.0)

Jin et al. [5]	2006	9	Henle's trunk (88.9)
			Flow into SMV with ASPDV (11.1)

Sakaguchi et al. [10]	2010	102	Henle's trunk (77.5)
			SMV (22.5)

Miyazawa et al. [12]	2015	100	Henle's trunk (93.0)
			Flow into SMV with ASPDV (7.0)

Cao et al. [21]	2015	144	Henle's trunk (75.0)
			Flow into SMV with ASPDV (18.8)
			SMV (6.3)

Lee et al. [9]	2016	116	Henle's trunk (79.3)
			Flow into SMV with ASPDV (16.4)
			SMV (4.3)

ASPDV = anterior superior pancreaticoduodenal vein; RGEV = right gastroepiploic vein; SMV = superior mesenteric vein.

**Table 7:** Types of draining pattern of the right side of the colon.

Author	Year	Case (*n*)	SRCV (%)	RCV (%)	MCV (%)	aMCV (%)
Yamaguchi et al. [3]	2002	58	N	Henle's trunk (19.0)	Henle's trunk (12.1)	Henle's trunk (39.7)
				SMV (24.1)	SMV (84.5)	SMV (29.3)
				Absent (43.1)	IMV (1.7)	Absent (25.9)
					SV (1.7)	

Jin et al. [5]	2006	9	Henle's trunk (88.9)	Henle's trunk (55.6)	Henle's trunk (11.1)	N
			Absent (11.1)	SMV (11.1)	SMV (88.9)	
				Absent (33.3)		

Sakaguchi et al. [10]	2010	102	Henle's trunk (74.5)	Henle's trunk (24.5)	Henle's trunk (19.6)	N
			SMV (15.7)	SMV (25.5)	SMV (80.4)	
			Absent (8.8)	Absent (50.0)		

Ogino et al. [11]	2014	81	Henle's trunk (21.0)	Henle's trunk (83.9)	Henle's trunk (19.8)	N
			Absent (79.0)	SMV (9.9)	SMV (67.9)	
				Absent (9.2)	JV (6.2)	
					IMV (4.9)	
					SV (1.2)	

Miyazawa et al. [12]	2015	100	Henle's trunk (93.0)	Henle's trunk (8.0)	Henle's trunk (13.0)	N
			Absent (7.0)	SMV (48.0)	SMV (84.0)	
				Absent (44.0)	Absent (3.0)	

Maki et al. [26]	2016	331	N	N	Henle's trunk (29.3)	N
					SMV (62.5)	
					IMV (4.8)	
					JV (0.6)	
					SV (2.7)	

Lee et al. [9]	2016	116	N	SMV (19.0)	Henle's trunk (3.4)	Henle's trunk (1.7)
				Absent (81.0)	SMV (93.1)	SMV (22.4)
					SV (3.4)	SV (5.2)
						Absent (70.7)

Alsabilah et al. [8]	2017	70	N	Henle's trunk (24.3)	Henle's trunk (5.7)	Henle's trunk (7.1)
				SMV (18.6)	SMV (94.0)	SMV (8.6)
				Absent (57.1)		Absent (84.3)

RCV = right colic vein; MCV = middle colic vein; aMCV = accessory middle colic vein; SRCV = superior right colic vein; SMV = superior mesenteric vein; JV = jejunal vein; IMV = inferior mesenteric vein; SV = splenic vein; N = not mentioned.
